# Novel Computational Model for Recapitulating Patent Ductus Arteriosus Stenting Interventions

**DOI:** 10.1007/s00246-025-03909-2

**Published:** 2025-06-12

**Authors:** Luis René Mata Quiñonez, Leon Cheng, Andrew Marini, Srujana Joshi, Shweta Karnik, Lakshmi P. Dasi, Holly D. Bauser-Heaton

**Affiliations:** 1 Wallace H. Coulter Department of Biomedical Engineering at Georgia Institute of Technology and Emory University, Atlanta, GA USA; 2 Children’s Healthcare of Atlanta (CHOA), Atlanta, GA USA

**Keywords:** PDA stenting, Physics-based computational framework, Pre-procedural planning, Patient-specific data, Stent deployment, Predictive platform

## Abstract

Patent Ductus Arteriosus (PDA) stenting is a minimally invasive procedure used to maintain pulmonary blood flow in patients with ductal-dependent cyanotic congenital heart disease. However, because of its high complexity, anatomic variability, and frequent need for reinterventions, improved pre-procedural planning is necessary. In this retrospective study, we developed a physics-based computational framework to simulate PDA stenting using patient-specific data. We applied this method to two patients from a single center: Patient 1 had a type I PDA with a single stent implantation, while Patient 2 had a type II PDA and underwent placement of two stents. We segmented pre-procedural CT scans, modeled guidewire tracking and the bent pre-deployment configurations of the angioplasty balloon and stent, and simulated stent deployment within patient-specific PDA anatomies. Quantitative validation against post-procedural segmentations showed an average distance error of less than 1 mm, demonstrating high accuracy in replicating real-world outcomes. The pipeline effectively captured key mechanical interactions among the stent, balloon, guidewire, and PDA, highlighting phenomena, such as PDA straightening, changes in diameter and orientation, and the displacement of surrounding vasculature during deployment. Future work will integrate semiautomatic tools to predict the best-suited procedural parameters, including stent length, diameter, positioning, and vascular access and predicted risk of complications. Ultimately, our goal is to develop a predictive platform that enhances clinical decision-making, optimizes procedural efficiency, and reduces complications and reinterventions, thereby improving outcomes for pediatric patients undergoing PDA stenting.

**Supplementary Information:**

The online version contains supplementary material available at 10.1007/s00246-025-03909-2.

## Introduction

**Fig. 1:**
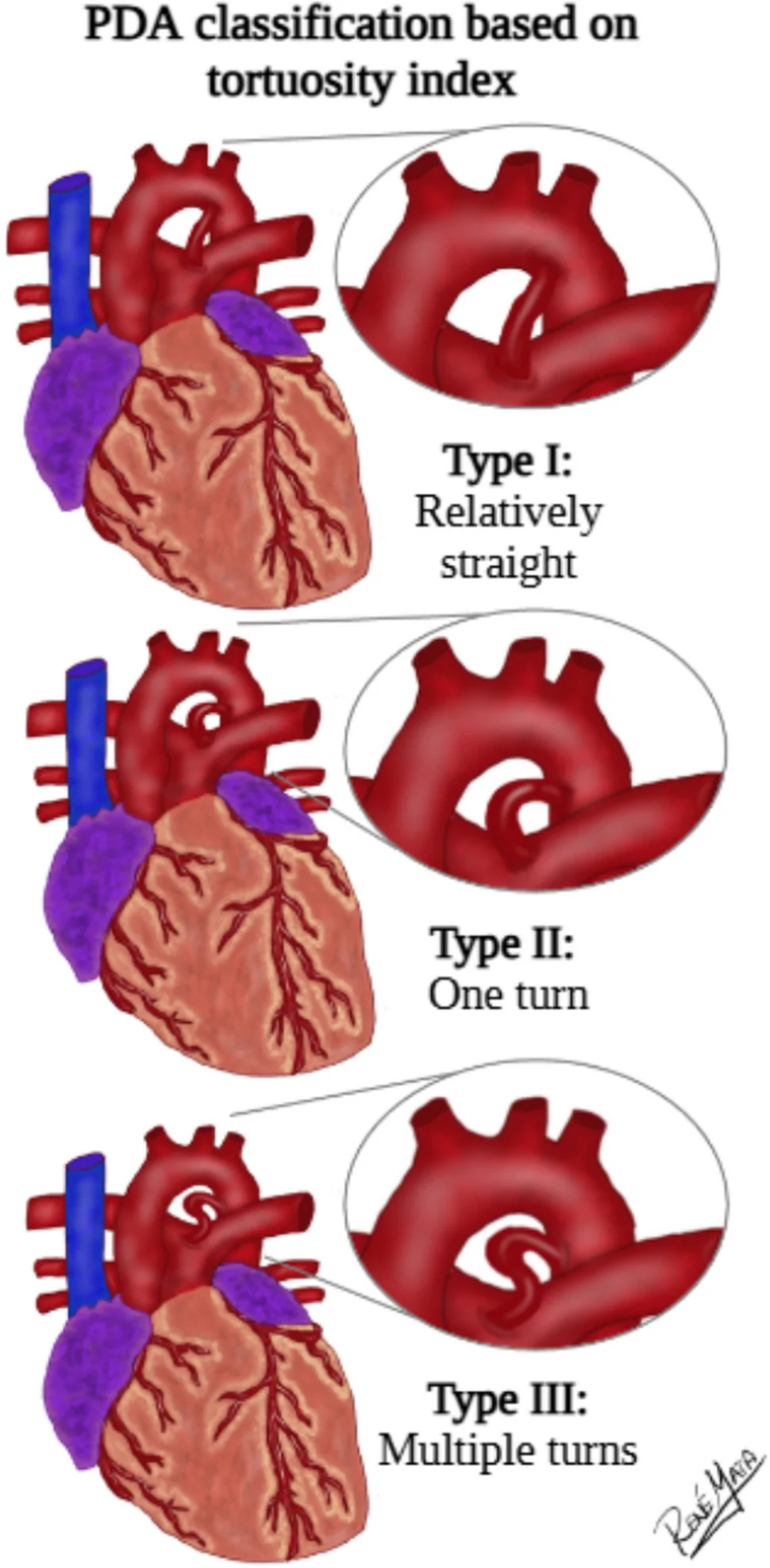
PDA tortuosity index can be categorized into three groups

Patients with ductal-dependent cyanotic congenital heart disease rely on an additional source of pulmonary blood flow (PBF) primarily through the PDA [1]. In typical development, this shunt obliterates after birth, isolating systemic and pulmonary circulations [2]. However, for these patients, preserving PDA patency is essential for PBF and survival. To utilize this natural shunt, clinicians perform PDA stenting (typically using Drug-Eluting Stents (DES) [3]), a minimally invasive palliative procedure that keeps the ductus open, improves oxygen saturation [4], and provides a more stable source of PBF [2]. PDA stenting offers a non-inferior and less invasive alternative to the gold-standard Blalock–Taussig–Thomas shunt (BTTS), a surgery that creates an artificial connection between an aortic branch and the pulmonary artery (PA) [1, 5–7]. While PDA stenting offers a lower mortality rate, hospital length of stay [1, 6, 7], risk of diuretic use at discharge, and duration of inotrope use [5]; four significant challenges overshadow the procedure: its complexity, planning methods highly reliant on clinician expertise [8, 9], high rate of interstage reinterventions [5], and the potential for downstream complications [8, 10]. The procedural complexity arises from the tortuous and highly variable anatomy of the PDA, which can have conical, tubular, or elongated shapes and originate from different locations of the aorta [11]. The tortuosity index classifies PDA anatomy into three types: type I, which is relatively straight; type II, which features a single curve; and type III, the “bizarre” configuration, characterized by two or more curves [12] (Fig. 1).

**Fig. 2:**
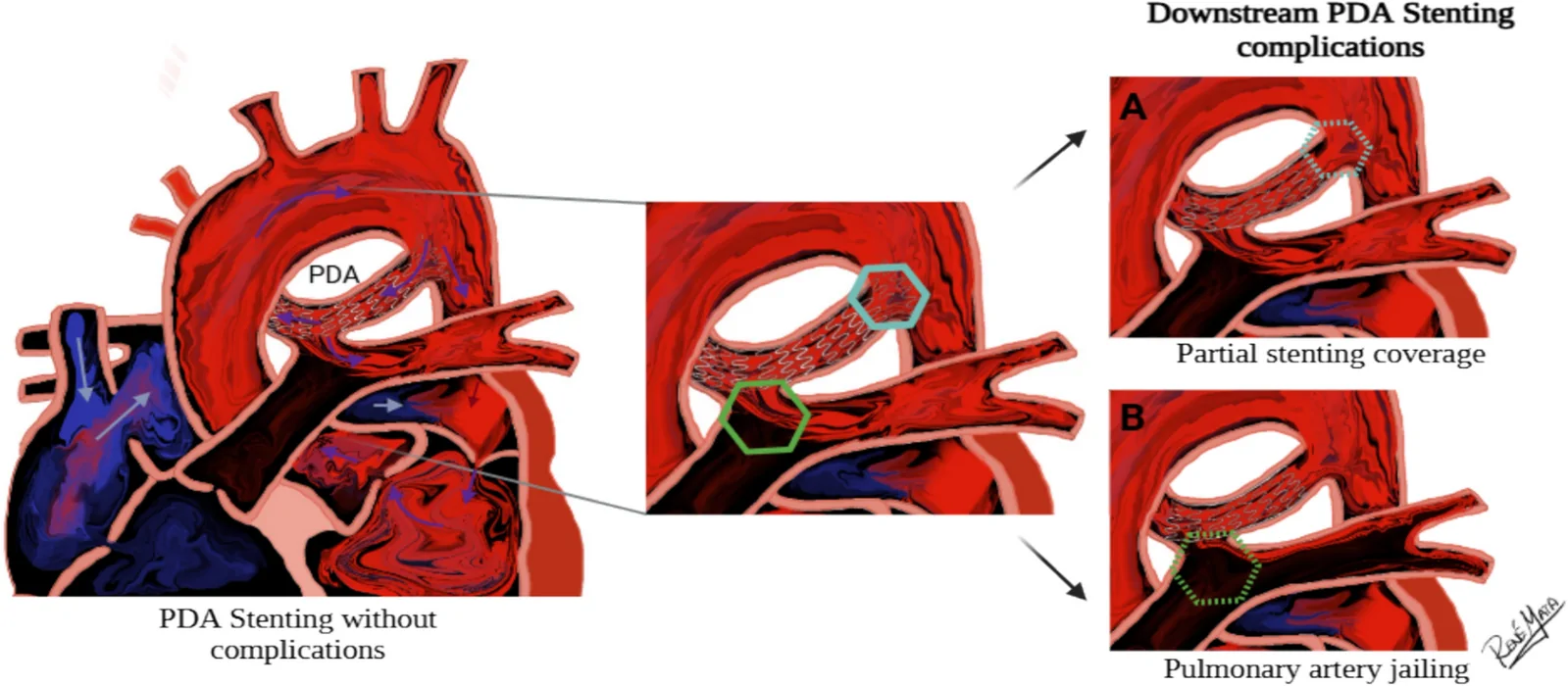
A successful PDA stenting procedure entails completely covering the PDA along its length with protrusions on the aortic and pulmonary sides and promoting a balanced PBF. The arrows indicate the blood flow in case of pulmonary atresia, where blue indicates low O_2_ saturation from venous circulation, red is oxygenated blood from systemic circulation, and purple is mixed venous and arterial blood. **A** During partial stenting coverage (dashed blue hexagon), either aortic or pulmonary segments are left unstented and are in risk of PDA stenosis and **B** PA jailing (dashed green hexagon) occurs due to blockage of the unstented side branch PA reducing the blood flow and PA growth

Anatomic variability complicates the prediction of the optimal strategy for proper stenting, necessitating a personalized approach [8, 13–15]. Compounding this challenge is the lack of standard and precise planning tools to select procedural stenting parameters. Although clinicians follow systematic protocols, current methods primarily rely on visual assessment and measurements from 2D angiography and 3D CT imaging to estimate PDA length, with stent diameter based on the patient’s weight and age [8, 9, 13]. Key decisions—such as catheter insertion access or stent length—are largely dependent on clinician expertise and often made in real-time during the procedure, leading to ad hoc adjustments [16–18]. Crucially, existing methods overlook the mechanical interactions among the stent, balloon, guidewire, and patient-specific anatomy, which can greatly influence outcomes. As a result, these limitations can contribute to downstream complications. In particular, inaccurate stent length selection may lead to partial stenting coverage of the PDA [9, 17] and PA jailing [1, 13], as illustrated in Fig. 2. A stent that is too short may leave segments of the PDA uncovered, accelerating stenosis due to the ductus’s natural obliterative process [19]. Conversely, PA jailing occurs when an excessively long stent obstructs the blood flow of the side branch PA [19], which often results in PBF imbalance and asymmetric PA growth [5, 13, 20]. These complications necessitate reinterventions in up to 50% of cases [5]. To address the complications, interventional cardiologists typically implant additional stents to either cover the exposed PDA segment, dilate intrastent neointimal proliferation, or perform side dilation and bifurcating stenting maneuvers to restore PBF [2, 5, 8].

**Fig. 3:**
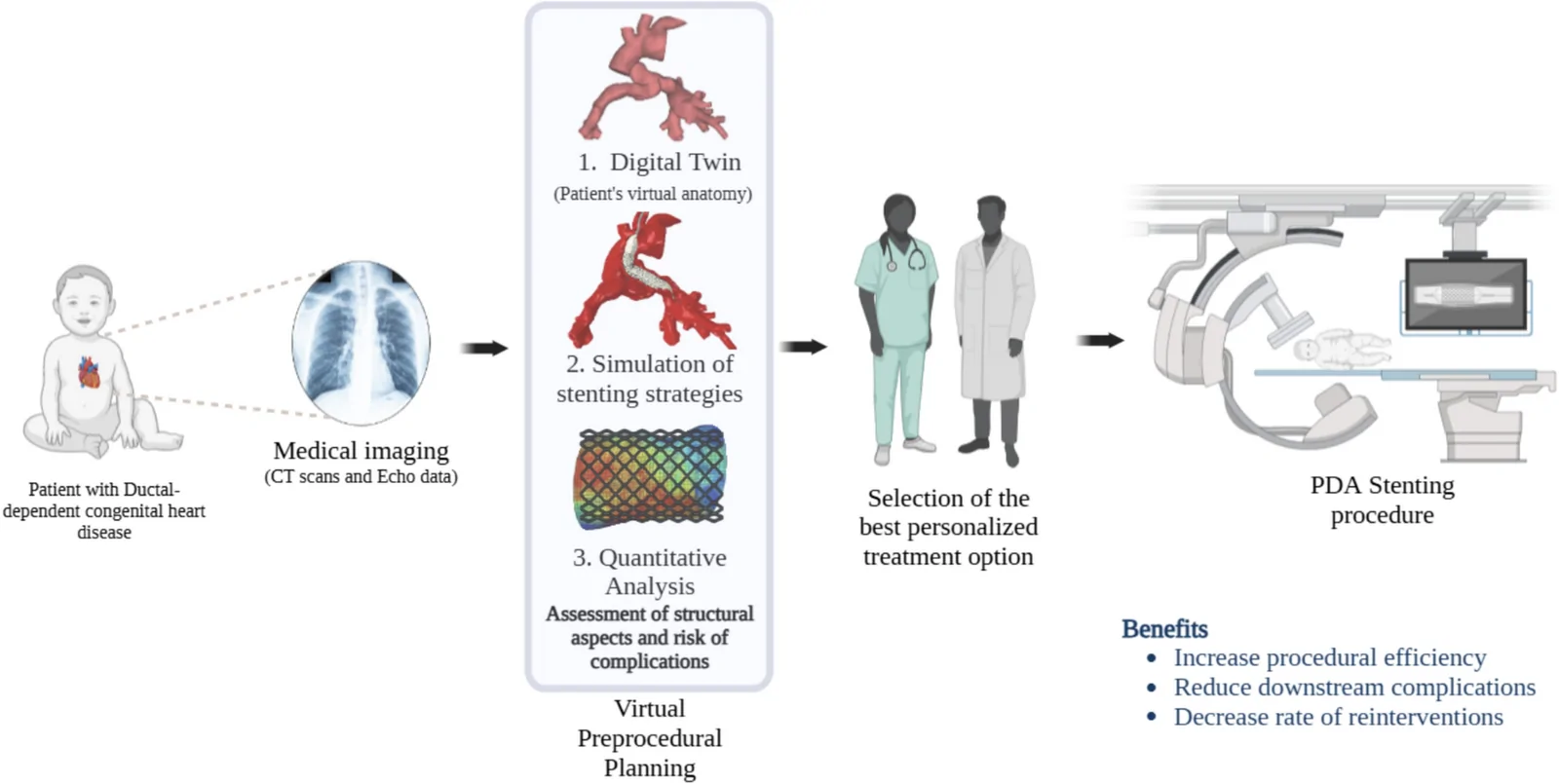
A pre-procedural planning platform that creates digital twins of patient-specific anatomy (3D models created from medical imaging that replicate an individual’s unique anatomic structure), allowing for the simulation and quantitative evaluation of multiple stenting strategies to predict complication risks, could assist clinicians in selecting the optimal treatment for each patient

Given these challenges, there is a critical need to improve pre-procedural planning for PDA stenting. Adopting a more quantitative approach is essential to guide clinical decision-making and optimize patient-specific stenting strategies. Our long-term vision is to develop a computational platform capable of analyzing potential clinical scenarios and delivering key insights—such as best-suited stent length and diameter, appropriate stent positioning and vascular access, and predicted risk of partial stenting coverage and PA jailing—all before the procedure. Having access to these insights in advance will equip cardiovascular teams with the tools to choose the best personalized treatment option, streamline procedural efficiency, and, most importantly, reduce complications and reinterventions to ensure better outcomes for patients (Fig. 3).

As the first step toward our goal, we present a retrospective computational framework grounded in physics-based simulations to model the PDA stenting procedure, utilizing patient-specific data from a single center. Our objective was to computationally reproduce the outcomes of the intervention and validate them against post-procedural CT scans. We created personalized *digital twins* (3D models that mimic the unique anatomic features of an individual) from imaging data and simulated key stages of the intervention. This included generating the stent and angioplasty balloon pre-deployment configurations based on guidewire placement, as well as simulating stent deployment along the PDA. Finally, we conducted a quantitative validation of our pipeline by estimating the error and evaluating the agreement between our simulated outcomes and actual post-procedural results. We computed the statistical distribution of distances between the vertices of the simulated stent and the segmented post-procedural stent, along with additional statistical and geometric descriptors.

## Methods

In clinical practice, pre-procedural planning for PDA stenting involves a collaborative effort among interventional cardiologists, radiologists, and other clinicians. Utilizing 3D CT reconstructions and measurements, they assess PDA morphology (primarily tortuosity and orientation), length, and diameter to have a better understanding of the medical devices to be used during the procedure [13]. In the catheterization laboratory, after anesthesia and catheter placement, a guidewire is advanced via transarterial access until it traverses the PDA [8]. The PDA length is measured again using frames of 2D angiographic images and decisions about the best-suited stent length are made. The selected stent and associated angioplasty balloon are then tracked along the guidewire and finally the stent is deployed [19].

**Fig. 4:**
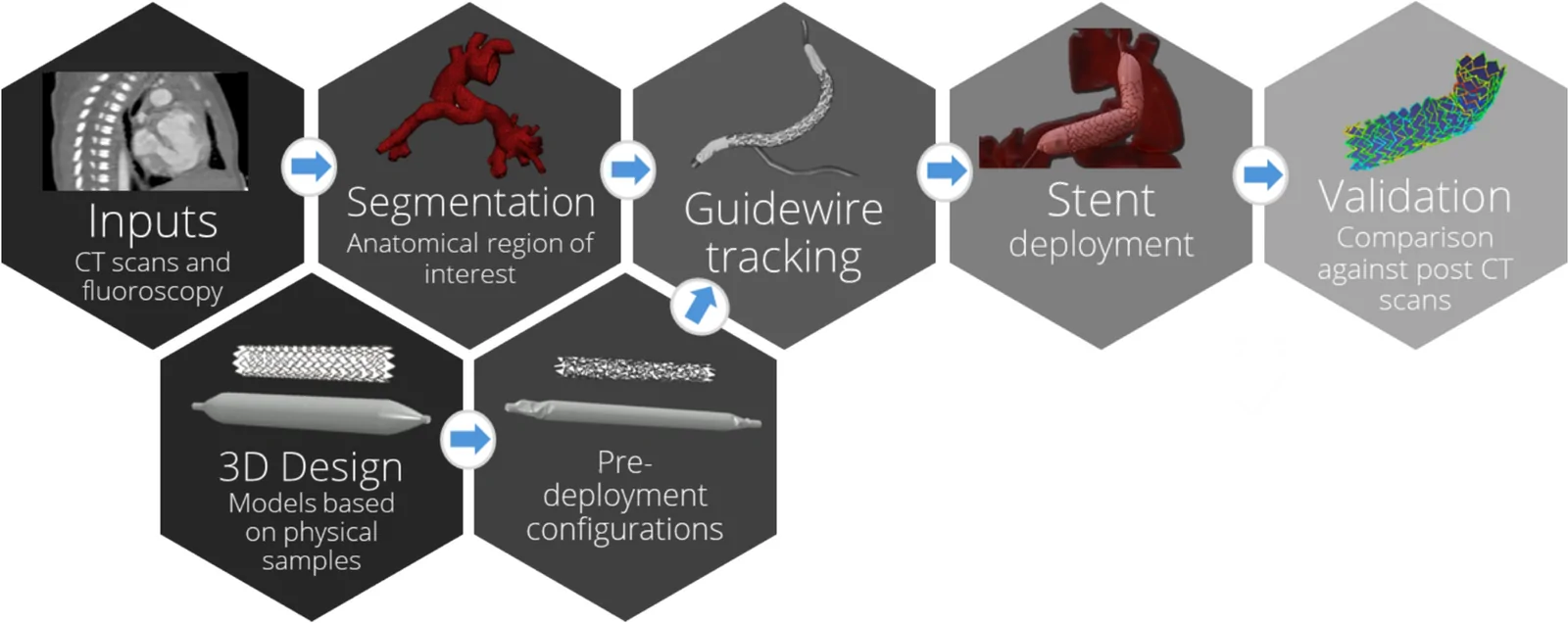
Our computational simulation framework of PDA stenting interventions reconstructs 3D models of patient’s anatomy through segmentation. Simultaneously, computational 3D models of stent, and angioplasty balloon (based on scans of physical samples) are created and radially crimped to obtain the pre-deployment configurations. These models are bent and positioned within the patient’s anatomy according to the guidewire pathway. We conduct physics-based simulations to model the inflation and deflation of the angioplasty balloon and the complex mechanical interactions that drive stent deployment. Finally, the simulations are validated by comparing the results with post-procedural segmentations from real interventions

To model and replicate the PDA stenting procedure, we developed a pipeline integrating patient-specific segmentation, Computer-aided Design (CAD)-based 3D modeling of devices, physics-based simulations, and mesh transformations to simulate guidewire placement and stent deployment. Figure 4 provides an overview of the method, with each step described in detail below. This simulation pipeline aims to characterize the structural aspects of the intervention, detailing how different elements physically interact and contribute to stenting outcomes.

### Inputs: Imaging Data

In this retrospective study, we applied our computational framework to two patients who underwent PDA stenting. We collected patient data, including pre- and post-procedural CT scans, procedural fluoroscopy, and physical samples of the same models of stents and angioplasty balloons used in these interventions. A Data Usage Agreement (DUA) between Children’s Healthcare of Atlanta (CHOA) and the Georgia Institute of Technology, approved by the Institutional Review Board (IRB), ensured compliance with the Health Insurance Portability and Accountability Act (HIPAA) to protect patient confidentiality.

### 3D Segmentation

**Fig. 5:**
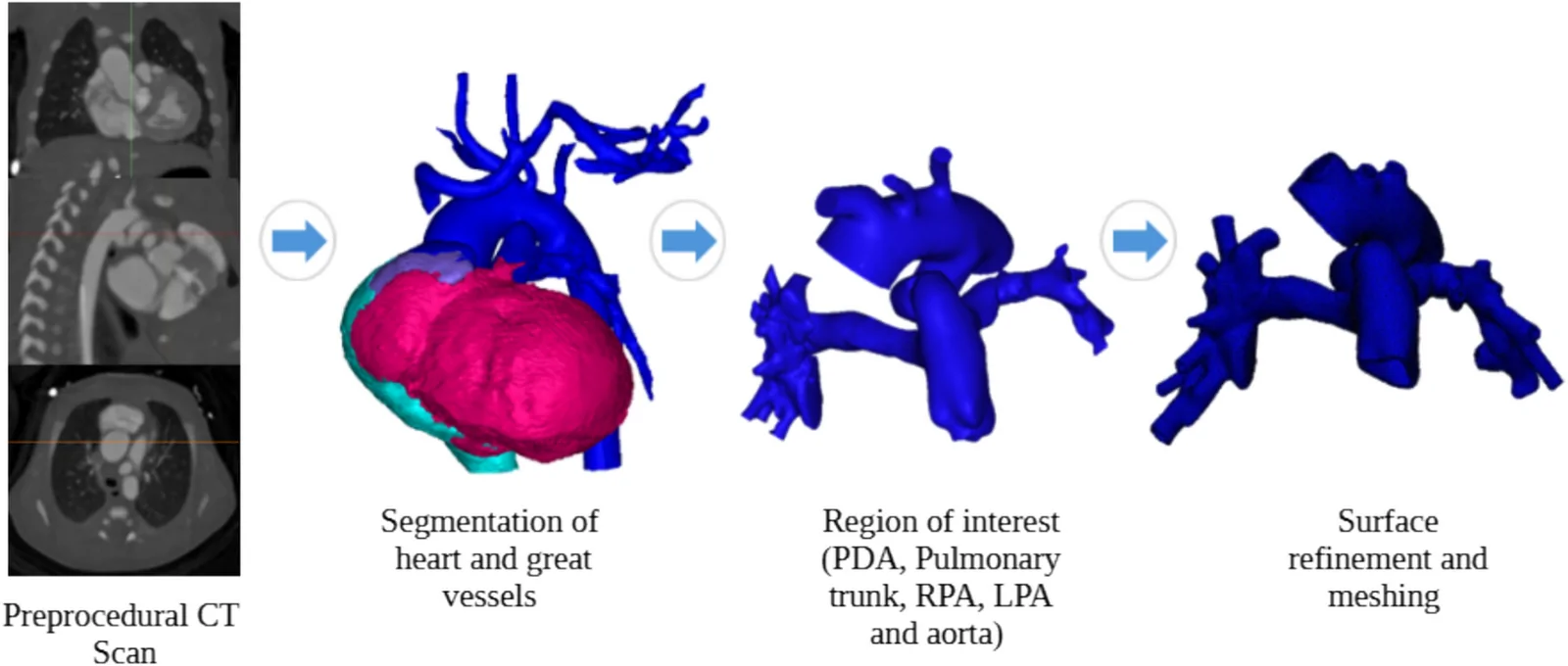
CT scans were segmented, ROIs isolated, surface refined, and mesh created. The resulting mesh was used for stent deployment simulations

Our initial processing step involved computationally reconstructing the regions of interest (ROIs) of patient’s anatomy from pre-procedural CT scans, including the PDA and adjacent structures such as the descending aorta, aortic arch, and its branches, including proximal sections of the brachiocephalic trunk, left and right main carotid and subclavian arteries, as well as the main, left, and right pulmonary arteries and their bifurcations near the lung hilum. Mimics software (Materialise, Leuven, Belgium) was utilized to extract these vascular structures of interest based on their voxel intensity. We then refined the resulting geometries using slice and mask editing techniques. The 3D-reconstructed models were further polished and discretized in 3-Matic (Materialise, Leuven, Belgium) to generate meshed geometries for simulations (Fig. 5).

### CAD Modeling

**Fig. 6:**
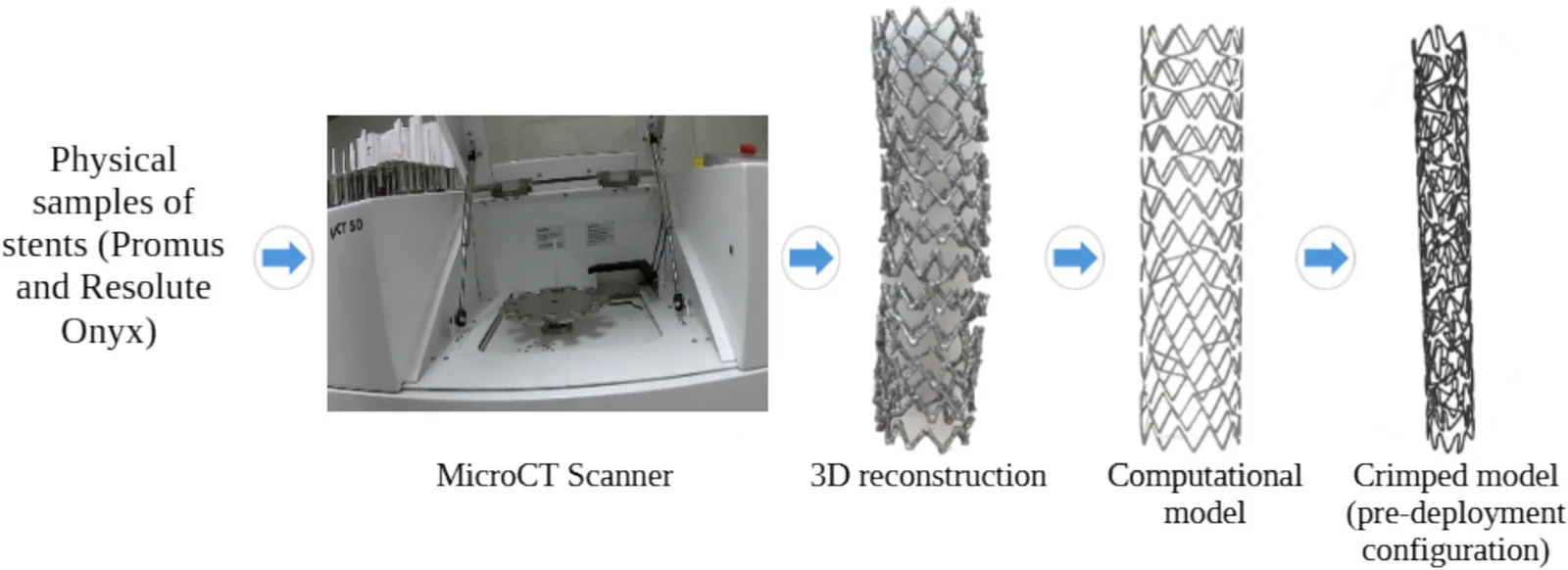
Computational stent models were created using measurements from 3D-reconstructed micro-CT scans of physical samples using the uCT 50 micro-CT scanner (Scanco Medical, Brüttisellen, Switzerland). The resulting geometry was then crimped through radial compression to achieve the pre-deployment configuration

We generated models of two different stent platforms: Promus Elite (Boston Scientific, Marlborough, Massachusetts, USA) [21] and Resolute Onyx (Medtronic, Minneapolis, Minnesota, USA) [22]. Using Solidworks, as shown in Fig. 6, we created bespoke models based on measurements obtained from micro-CT scans using the uCT 50 micro-CT scanner (Scanco Medical, Brüttisellen, Switzerland). We then discretized these geometries through meshing using the specialized software Hypermesh (Altair, Troy, Michigan, USA).

The angioplasty balloon design was created based on measurements from physical devices and sketched in the software Abaqus (Dassault Systemes, Vélizy-Villacoublay, France). Table 1 below shows the stent platform, diameter, and length of the devices used for each patient (note that two stents were implanted for patient 2).

**Table 1:** Stent platform and sizes employed during the PDA stenting procedure

Patient ID	Stent platform	Stent diameter	Stent length
1	Promus	4 mm	20 mm
2	Promus	4 mm	20 mm
	Promus	4 mm	8 mm

We then simulated the compression of the angioplasty balloon and crimping of the stent to obtain the default configurations before stenting. Individually (for stent and balloon), we executed a dynamic simulation using Abaqus (Dassault Systemes, Vélizy-Villacoublay, France) Explicit by radially contracting an outer cylinder through application of displacement boundary conditions. The cylinder deforms the stent and balloon until obtaining the crimped nominal diameters according to device specifications.

### Stent and Balloon Bending

The deformed guidewire pathways were extracted from the procedural fluoroscopy, and after postprocessing, filtering, and smoothing operations, they were used to accurately track the stent and angioplasty balloon to their correct pre-deployment positions along the PDA. To efficiently generate the pre-deployment deformed geometries of the stent and angioplasty balloon, we applied mesh transformations in Blender (Amsterdam, the Netherlands), minimizing geometric twisting.

### Stent Deployment

After integrating the patient-specific discretized anatomy with the pre-deployment crimped configurations of the stent and angioplasty balloon, aligned along the PDA, we assigned the material properties to each element of the framework and modeled the inflation and deflation sequences of the angioplasty balloon. For material properties of PDA, we employed a third-order hyperelastic Ogden model, derived from experimental data [23], which accurately captures the mechanical properties of vascular arterial tissue. We assigned a calibrated elasto-plastic model for the stent to mimic the material properties of platinum–chromium alloy in the Promus Elite stent [21, 24] incorporating isotropic hardening, i.e., the uniform expansion of the material’s yield surface in all directions during plastic deformation [25], which allows the stent to remain its expanded shape even after the applied forces are removed during the balloon deflation. We considered the material of the angioplasty balloon as elastic neo-Hookean with characteristics similar to Nylon [26], and for the stent guidewire, we used an elasto-plastic model describing stainless steel mechanical properties [27]. We represented the balloon as a fluid cavity with negligible initial volume applying a fluid exchange boundary condition to simulate the dynamics of the incoming fluid, which consists of physiological solution (0.9% NaCl) with contrast medium, following a constant volumetric flow rate. Balloon inflation and deflation were controlled by adjusting the amplitude to match the required volume changes. We also included a contact interaction with friction-penalized tangential and hard normal contact behaviors. This allowed us to simulate the complex mechanical interactions occurring among the angioplasty balloon, stent, guidewire, and PDA.

### Validation

We segmented the stented PDA anatomy from post-procedural CT scans and overlaid it with the simulation results. This superposition was performed in MeshLab (Consiglio Nazionale delle Ricerche, Rome, Italy) using a process to overlay both simulated and segmented stents to ensure the maximum overlap between the volumes. We applied a filter to measure the distances between corresponding vertices in each geometry and analyzed their statistical distribution, considering both positive and negative values. Heatmaps and histograms were created to visualize the distance distribution for each mesh and the group as a whole. Two measurements were taken, each using a different geometry as the reference. We conducted normality analysis on this data and derived geometric metrics that quantitatively describe the shape of compared stents, including the dimensions of the bounding box surrounding the geometry, surface area, and principal axes.

## Results

### Patient Anatomic Segmentation, CAD Modeling, and Default Crimped Configurations for Both Patients

We successfully extracted the anatomic ROIs from patient-specific imaging data. The most challenging areas to delineate were the pulmonary arteries near the left atrium, but we accurately captured this anatomy using filtering and smoothing techniques. Table 2 presents the aortic and pulmonary origins of the PDA, along with its tortuosity index classification based on pre-procedural CT scans. Additionally, we measured patient-specific PDA characteristics, including length, minimal diameter, average cross-sectional area, and average ellipticity (where a value closer to one indicates a more circular shape and a value closer to zero represents a more elongated ellipse). The PDA morphology in patient 2 was more complex than in patient 1 due to its pronounced curvature (type II), whereas patient 1 exhibited greater variation in diameter. Despite these differences, both anatomies had similar cross-sectional areas. Based on ellipticity values, the PDA in patient 1 was significantly more circular compared to that of patient 2.

**Table 2:** Unique features of patient’s PDAs included in this study

Patient ID	PDA Aortic Origin	PDA Pulmonary Origin	Classification based on tortuosity	Length	Minimal diameter	Diameter standard deviation	Average cross-sectional area	Average ellipticity
1	Descending aorta	Proximal segment of right PA (RPA)	Type I (Relatively straight)	13.22 mm	1.43 mm	0.7745 mm	34.78 mm^2^	0.8271
2	Descending aorta	Distal segment of main PA (MPA)	Type II (Curved)	16.13 mm	2.66 mm	0.6940 mm	33.13 mm^2^	0.5475

Regarding the CAD models of the Promus and Onyx stents, we obtained clean meshes with a combination of hexahedral and tetrahedral elements; however, in this case, we only used the Promus model since this platform was employed in the analyzed patients. The crimping simulations successfully replicated the default configurations of the stents as observed upon removal from their packaging.

### Guidewire Tracking, Stent Deployment, and Validation Results

#### Patient 1

**Fig. 7:**
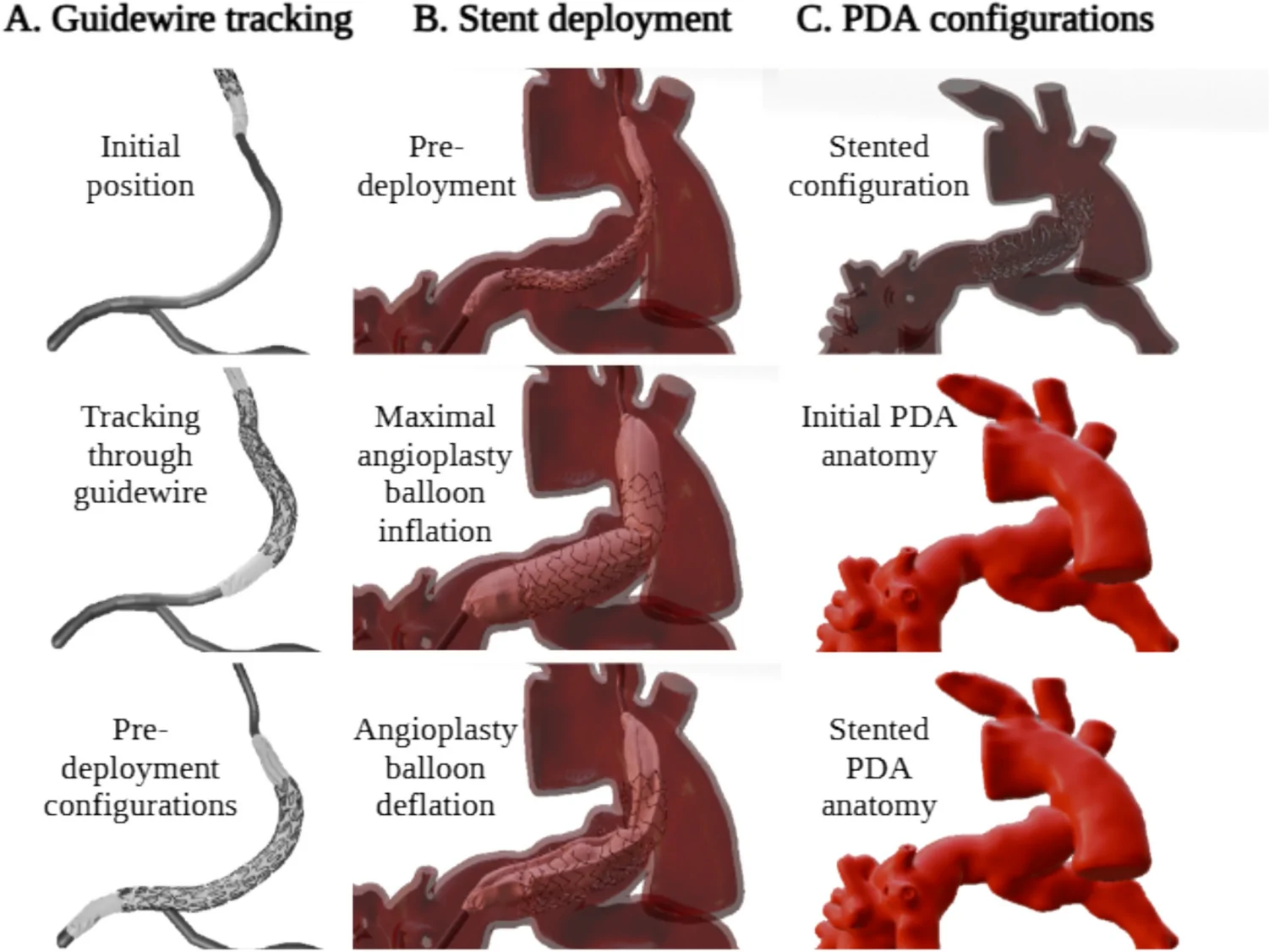
**A** Our results of the guidewire tracking simulation, stent and balloon bending, and positioning of the pre-deployed configurations along the PDA. **B** Key stages of the dynamic stent deployment simulation, including the initial position (pre-deployment geometries), maximal angioplasty balloon inflation, and the final step after deflation. **C** The resulting deployed stent acquired a curved cylindrical shape with variable diameter completely covering the PDA along its length, including aortic and pulmonary protrusions of 5 and 3 mm, respectively. When comparing the initial and stented anatomies, we observed a more vertical PDA orientation and increased diameter in the aortic origin

We successfully extracted the guidewire contour and reconstructed its 3D trajectory using centerline analysis and smoothing techniques. The angioplasty balloon and stent were aligned and positioned along this path using mesh transformation in Blender. In Fig. 7, panel A shows the results of our guidewire tracking simulation, where we captured three different frames: the initial position, mid-way tracking, and the final position representing the pre-deployment configuration of the bent stent and angioplasty balloon. In panel B, we present specific frames of our dynamic stent deployment simulation. The pre-deployment phase shows the guidewire, stent, and angioplasty balloon positioned within the PDA, introduced via the left carotid artery. The 4 × 20 mm stent was placed with an aortic protrusion of 5 mm and a right pulmonary protrusion of 3 mm. During maximal balloon inflation, the stent expanded, interacting with the PDA lumen and slightly shifting to a more vertical orientation. The guidewire provided support to prevent migration, keeping the stent in position throughout the deployment process. However, minor tilting and oscillatory movement in the anterior–posterior direction were observed. After deflation, the stent remained in place through plastic deformation and isotropic hardening. Minimal recoil was observed, as indicated by the maintained aortic and pulmonary protrusions and the limited displacement of the stent during the deflation phase. In panel C, the initial and stented PDA configurations are shown. The deployed stent conformed to the natural curvature of the PDA, assuming a curved cylindrical shape with a variable diameter, particularly in the proximal segment. The final images highlight the external surfaces of the PDA before and after stenting, demonstrating notable dilation at the aortic origin.

**Fig. 8:**
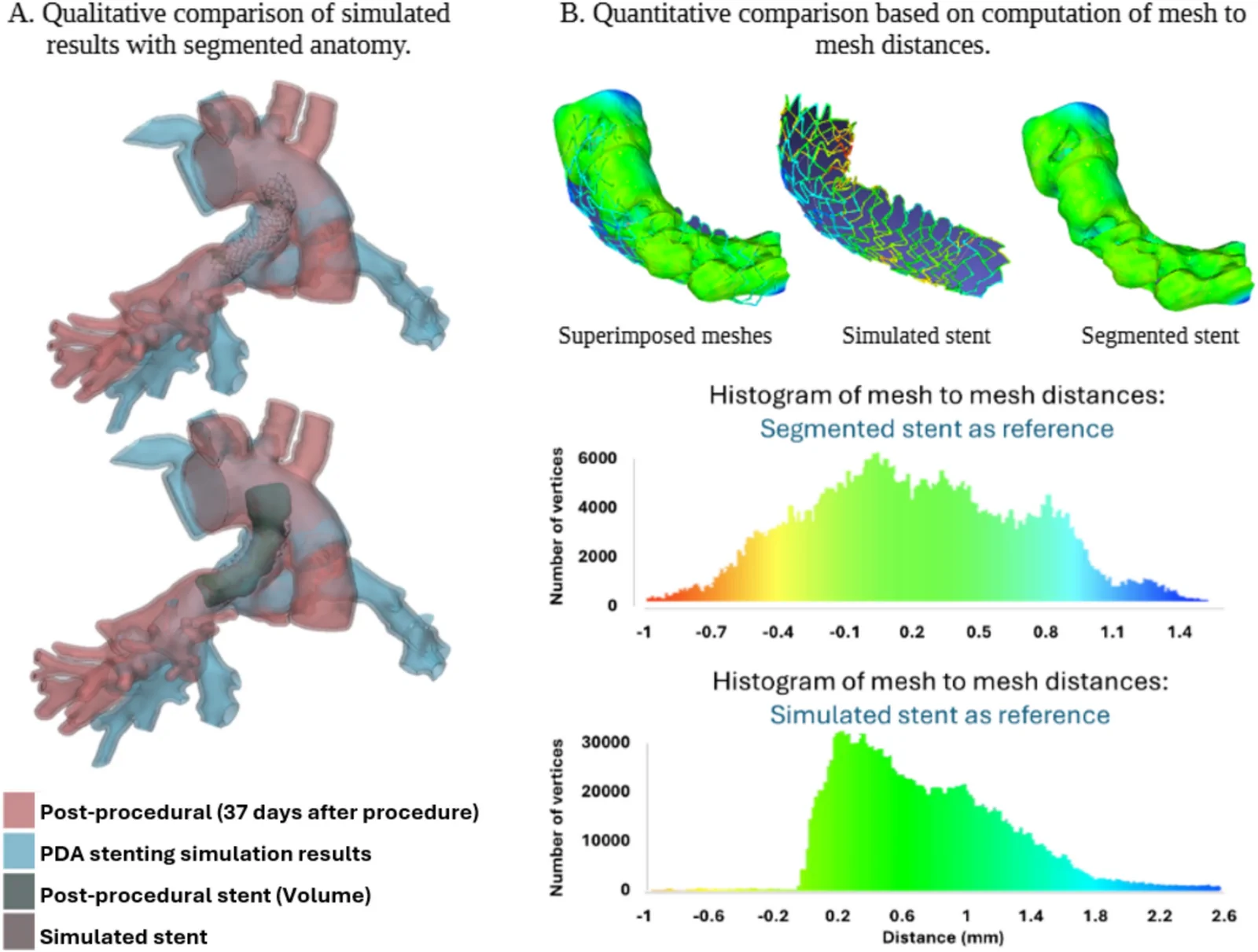
**A** Superposition of the simulated anatomy with segmentations from postprocedural CT scans to compare the PDA stented configurations. **B** Quantitative validation comparing the distances between simulated and segmented stents shows a non-normal distribution with an average distance of 0.087 mm, median value of 0.06481 mm when the segmented stent was the reference and average of 0.8103 mm and median of 0.7138 mm with simulated stent as baseline. We also measured the dimensions of the resulting bounding box, surface area, and principal axes

In Fig. 8, panel A, we present an overlay of the simulated post-stenting PDA anatomy and the post-procedural segmentation. Despite observable differences in vascular structures due to the patient’s natural growth over the 37-day period between the procedure and CT scan acquisition, the stented PDA remains relatively unchanged except for the adjacent remodeling. PA growth is evident, whereas the aortic arch and supra-aortic branches show minimal variation. During segmentation, we were unable to capture the finer details of the stent, obtaining instead a porous volume that delineates its shape. This amorphous structure may result from post-procedural remodeling driven by neointimal proliferation, primarily mediated by macrophages, endothelial and smooth muscle cells [28]. Due to the similar radiopacity, the resulting segmentation includes this additional layer of surrounding tissue. However, due to the stent’s stiffness and radial strength, the dimensions of the stented region remain largely stable, making it a reliable reference for validation. In the supplementary material, Video 1, we provide a video animation of our simulation results for patient 1.

In panel B, the top image shows the superposition of the compared stents, followed by individual views of the simulated and segmented stents placed side by side. A color-coded filter highlights the signed distances between the two stents (positive or negative, depending on the chosen reference). To visualize these distances, we generated two histograms displaying their distribution in millimeters, calculated across the mesh vertices. The first histogram shows distances with the simulated stent as the reference, while the second uses the baseline stent. In both histograms, the y-axis represents the number of vertices, and the x-axis indicates the measured distance. Both measurements were performed to provide a more accurate representation of the differences between the two geometries.

Normality analysis of histogram 1 using the Shapiro–Wilk and Anderson–Darling tests revealed that the data are not normally distributed (Shapiro–Wilk Test Statistic: 0.9354, Anderson–Darling Test Statistic: 6261.6737, *p* = 0.0000, *α* = 0.05). Similar analysis for the second histogram indicates a non-normal data distribution (Shapiro–Wilk Test Statistic: 0.9168, Anderson–Darling Test Statistic: 3585.1675, *p* = 0.0000, *α* = 0.05). Table 3 presents the statistical results of the measured distances.

**Table 3:** Statistical parameters describing the distribution of mesh-to-mesh distances in Patient 1

Metric	Histogram 1: Segmented stent as reference	Histogram 2: Simulated stent as reference
Average	*0.087788* mm	*0.810327* mm
Median	*0.064819* mm	*0.713847 *mm
Minimum value	− 0.933647 mm	− 1.938743 mm
Maximum value	1.506836 mm	3.290582 mm
Standard deviation	0.419037 mm	0.614666 mm
Variance	0.175592 mm^2^	0.377814 mm^2^

Italic values represent most reliable indicators of error for validation purposes

The average and median values indicate that *both geometries are similar, with an error of less than 1 mm*. However, the segmented stent was approximately 2 mm longer than the simulated one, particularly on the aortic side, which could bias the statistical results. This discrepancy may be due to the implanted stent exceeding the nominal 20 mm length. The mesh bounding box enclosing both overlaid stents measured 16.2636 mm in length, 8.4090 mm in width, and 11.6875 mm in height, with a total surface area of 123.7108 mm^2^. The principal axes of the mesh are (0.4313, − 0.2675, 0.861), (0.6434, 0.7606, − 0.0859), and (0.6324, − 0.5914, − 0.5002).

#### Patient 2

Patient 2 underwent multi-stent implantation, with a second stent deployed during a reintervention to cover a small unstented region at the aortic origin of the PDA mitigating the risk of PDA stenosis. The simulation process began with guidewire tracking and deployment of the first stent. Using the resulting geometries of the stented PDA from this initial deployment, we simulated the tracking and placement of the second stent.

**Fig. 9:**
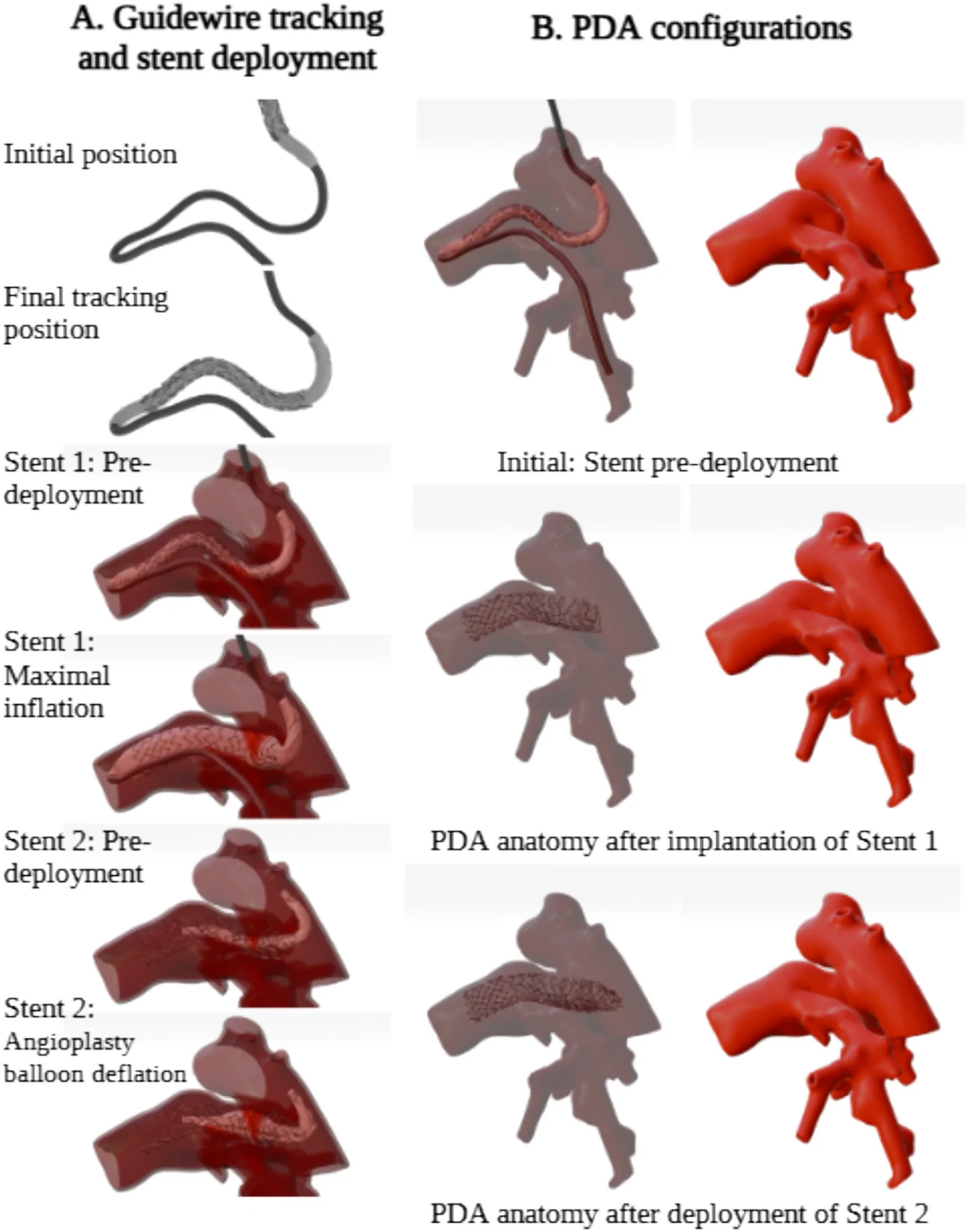
**A** Stages of dynamic simulation results for this multi-stent case, including guidewire tracking and stent deployment for stents 1 and 2. B Initial and stented configurations of the PDA after stents 1 and 2, notice the change in conformation from a curved to a relatively straight geometry and the change in aortic protrusion and stent overlapping after the implantation of the second stent

In Fig. 9, panel A, we illustrate key steps of the process. These include the initial and final guidewire tracking positions for the first stent, the pre-deployment configuration, and maximal inflation. We also show the final tracking position for the second stent and the final frame of angioplasty balloon deflation following the deployment of the second stent. In Fig. 9, panel B, we present the initial PDA configuration and the PDA anatomy following the deployment of the first and second stents. Unlike Patient 1, the PDA did not undergo significant reorientation but was displaced superiorly over the MPA. This type II PDA had a sharp inferiorly convex curve, which, after the implantation of both stents, transformed into a relatively cylindrical shape. During the deployment of the second stent, PDA displacement was minimal due to the rigidity of the first stent. The initial stent (4 × 20 mm) was positioned 1 mm below the aortic origin of the PDA and extended into the MPA, posing a risk of ductal stenosis if left unstented [19]. To address this, a second stent (4 × 8 mm) was placed with a 3-mm protrusion on the aortic side, covering the previously unstented segment and overlapping the first stent distally by approximately 4 mm. The misalignment of the first stent was likely influenced by the sharp angle between the aorta and the PDA’s aortic origin, a consequence of the PDA’s horizontal orientation, which prevented proper anchoring of the stent’s inferior edge at the aortic origin. Due to the stent’s radial strength, minimal recoil was observed, and the resulting PDA acquired a moderately cylindrical geometry with a variable radius, as the stent partially conformed to the elliptical shape of the original PDA. Additionally, the diameter of the second stent was smaller than that of the first, likely due to the constraint imposed by the first stent and the sharp proximal curve of the angioplasty balloon, which was in close contact with the posterior wall of the aortic arch. Refer to the supplementary material, Video 2, for a video animation of our simulation results for patient 2.

**Fig. 10:**
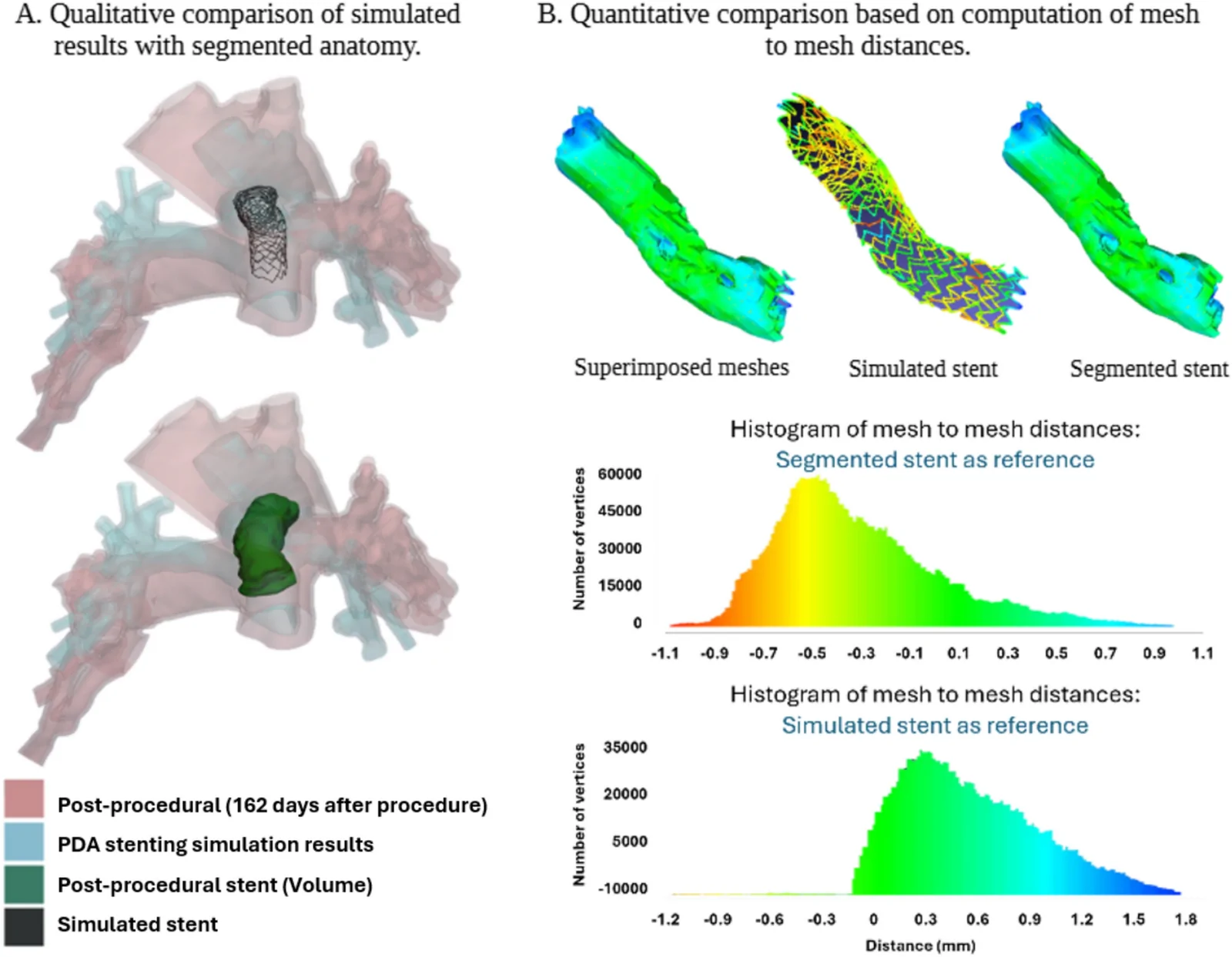
**A** In this superposition of simulated and post-procedural anatomies, we appreciate a significant difference in vascular size while the stented PDA region is similar. **B** Quantitative validation comparing the distances between simulated and segmented stents shows a non-normal distribution with an average distance of − 0.2384 mm, median value of − 0.3018 mm, with a range from − 1.002 to 1.25 mm based on segmented stent as reference. When the simulated stent was considered as baseline, the average distance was 0.6014 mm, median value of 0.5443 mm, ranging from − 1.6824 to 1.9186 mm. As in the previous case, the dimensions of the resulting bounding box, surface area, and principal axes were computed as well

For validation, we followed the same approach as in the previous patient, overlaying our simulation results with the segmentation of post-procedural CT scans. In this case, the CT scans were acquired 162 days after the procedure, revealing significant changes in vascular structures, as shown in Fig. 10, panel A. However, the stented PDA remained relatively unchanged, allowing us to use it as a baseline for validation.

In panel B, we present the results of quantitative validation, comparing the signed distances between the vertices of the simulated and post-procedurally segmented stents. The corresponding histograms illustrate the statistical distribution of these distances considering in each case the segmented stent and simulated stent as baselines, respectively. Statistical assessment for normality using the Shapiro–Wilk and Anderson–Darling tests for the first histogram confirmed that the data is not normally distributed (Shapiro–Wilk Test Statistic: 0.9879, Anderson–Darling Test Statistic: 259.9098, *p* = 0.0000, α = 0.05). Similar results were found for the second case. Data are not normally distributed (Shapiro–Wilk Test Statistic: 0.9366, Anderson–Darling Test Statistic: 3399.7887, *p* = 0.0000, α = 0.05). In Table 4, we present the statistical results for the analysis of both distributions.

**Table 4:** Statistical parameters characterizing the distribution of mesh-to-mesh distances in Patient 2

Metric	Histogram 1: Segmented stent as reference	Histogram 2: Simulated stent as reference
Average	− *0.238375* mm	*0.601442* mm
Median	− *0.301854* mm	*0.544336* mm
Minimum value	− 1.001991 mm	− 1.682443 mm
Maximum value	1.250031 mm	1.918581 mm
Standard deviation	0.313564 mm	0.390647 mm
Variance	0.098322 mm^2^	0.152605 mm^2^

Italic values represent the most reliable indicators of error for validation purposes

As in the previous patient, *the error distances were less than 1 mm, as indicated by the average and median values*. We found more balanced distributions in this case, with greater variability on the aortic side compared to other segments of the stent. The bounding box enclosing both overlaid meshes measured 21.21 mm in length, 9.79 mm in width, and 7.24 mm in height, with a total mesh surface area of 187.10 mm^2^. The principal axes were oriented along (− 0.6028, − 0.7529, 0.2640), (− 0.0429, − 0.2998, − 0.9530), and (0.7968, − 0.5858, 0.1484).

## Discussion

In this retrospective study, we created a computational simulation pipeline for PDA stenting, achieving a high degree of similarity when compared with real-world outcomes. Our quantitative validation, considering post-procedural stent segmentations, *demonstrated average distance errors lower than 1 mm for patients 1 and 2* (0.0878 and 0.8103 mm. in patient 1 and − 0.2384 and 0.6014 mm for patient 2 considering the segmented and simulated stents as reference points for each case, respectively). Deviations were most pronounced in the highly tortuous regions and near the aortic origin, primarily due to diameter differences from post-stenting remodeling caused by neointimal proliferation, as well as the sharp curvature of the angioplasty balloon, which increased resistance to inflation on the aortic side. In patient 1, the segmented stent measured approximately two millimeters longer than its nominal length. By incorporating guidewire tracking and stent deployment in complex, patient-specific anatomies, our pipeline captured subtle changes in the orientation and diameter of the PDA and surrounding structures. Generally, we observed PDA straightening due to the stent’s rigidity, maintaining its expanded position through plastic deformation and isotropic hardening phenomena [25], but at the same time adapting to the organic curvatures of the PDA anatomy. The aortic origin and proximal regions of the pulmonary arteries also experienced displacement during deployment due to the stent’s residual radial force.

Our literature review identified multiple approaches aimed at improving pre-procedural planning for PDA stenting and enhancing the understanding of post-procedural complications. These contributions can be categorized into three main groups: (1) the use of machine learning methods to characterize key risk factors associated with PDA stenting, (2) leveraging Computed Tomography Angiography (CTA) to obtain measurements that guide procedural parameter selection, and (3) applying computational methods such as Computational Fluid Dynamics (CFD) and Finite Element Analysis (FEA) to assess stent hemodynamic performance and mechanical failure risk, respectively.

Yoon Na et al. compiled a list of risk factors in a large cohort of patients who underwent symptomatic and asymptomatic PDA stenting. Their study included 47 features covering prenatal conditions, delivery-related factors, and postnatal variables [29]. Machine learning algorithms were applied to predict the occurrence of complications after stenting [29]. While this study highlights the significance of perinatal factors in achieving successful outcomes, it overlooks the mechanical aspects of PDA stenting, which are directly linked to complications and reinterventions.

Efforts to enhance pre-procedural planning for this minimally invasive procedure are demonstrated in studies by Jadhav et al. [19], Graff et al. [30], and Chamberlain et al. [31]. Jadhav et al. conducted a study comparing the tortuosity index and PDA length measurements obtained from CTA and catheter angiography [19]. PDA length was assessed using the vessel centerline and compared against routine angiographic measurements [19]. Correlation analysis showed agreement in tortuosity index and aortic origin assessments but revealed poor agreement between the straight-line PDA length measured via catheter angiography and the eventual stented PDA length [19]. This discrepancy highlights the potential benefits of CTA in providing a more accurate estimation of PDA length. Graff et al. focused on identifying indicators related to the risk of PA jailing and predicting whether a patient would require PA intervention to mitigate this risk [30]. They found that the distance from the PA bifurcation to the largest diameter of the PA receiving the PDA serves as a strong predictor for the need for PA intervention [30]. Chamberlain et al. applied CTA to optimize the selection of vascular access for PDA stenting using 3D segmentations of patient-specific anatomies [31]. Their analysis involved 2D measurements of catheter angulation between the PDA’s aortic origin and potential vascular accesses, the degree of PA offset, and PDA tortuosity. They successfully guided vascular access selection for PDA stenting across varying tortuosity levels [31]. While these approaches provide valuable insights, they are susceptible to operator variability and inaccuracies in approximating 2D distances within complex 3D structures. More critically, they do not account for the essential stent–tissue interactions that result from the mechanical properties of materials and the dynamics of angioplasty balloon inflation and deflation interacting with the guidewire, stent, and patient-specific anatomy. These factors are essential for accurately modeling PDA stenting. Our approach provides a more comprehensive framework by considering the underlying physics, and with further refinement, we could derive insights into key procedural parameters.

Regarding the use of computational modeling techniques, Kori et al. developed a CFD framework to study the hemodynamic performance of stents and estimated parameters such as Wall Shear Stress (WSS), Time-Averaged Wall Shear Stress (TAWSS), and Oscillatory Shear Index (OSI) to predict the risk of in-stent restenosis [32, 33]. Their study combined experimental and computational methods, demonstrating that regions with low TAWSS (lower than 0.5 Pa) are prone to in-stent restenosis due to stagnation and the potential uptake of blood-borne particles [32, 33]. However, their analysis was based on simplified PDA geometries in different orientations (vertical or horizontal) and origins, without explicitly modeling the stent or its interaction with the anatomy. While WSS analysis is relevant for evaluating in-stent restenosis and thrombosis, our focus is on capturing personalized structural aspects of stent deployment, emphasizing the role of balloon, guidewire, and stent in determining the PDA’s final deformed configuration and prediction of complications.

Lazim et al. used FEA and experimental methods to identify zones of the stent prone to crack initiation and fracture due to fatigue stresses [34]. Their work explored various materials and strut configurations to optimize stent design [34]. However, their modeling approach was limited to straight anatomies, neglecting the pre-deployment bent configurations of the balloon and stent after guidewire tracking. Additionally, their inclusion of a plaque layer at the inner lumen close to the stent—despite its absence in patients requiring PDA stenting—along with the incorporation of elements specific to coronary artery disease, introduces limitations in clinical relevance. While this study is a foundational effort in investigating mechanical stent fracture, its primary objective is optimizing stent design rather than improving the pre-procedural planning of PDA stenting.

Meanwhile, numerous efforts have been made to computationally model stent deployment as a treatment for conditions like atherosclerosis, with approaches ranging from simplified models to extensive simulations that offer either structural or fluid dynamics analyses [35–37]. Our approach is the first computational physics-based model that accurately recapitulates real PDA stenting interventions. We introduced a distinct methodology by modeling the realistic shape deformations of the angioplasty balloon and stent according to the guidewire pathway thereby improving the accuracy of stent deployment simulations. Additionally, we leveraged Blender’s capability to simulate both balloon and stent bending, achieving significant reductions in simulation time compared to traditional methods.

Our future work focuses on improving the accuracy and clinical applicability of our model. Enhancing the simulation pipeline to better capture guidewire interactions with the PDA before deployment, refining angioplasty balloon expansion in sharply curved anatomies, and expanding our patient sample size will enable a more comprehensive assessment of procedural variability. We will advance predictive methodologies by analyzing simulation results to assess complication risks and determine optimal procedural parameters. To achieve this, we will integrate machine learning, optimization, and mathematical techniques to semi-automatically evaluate key procedural parameters, such as stent diameter, length, vascular access, positioning, and risks of partial stenting coverage and pulmonary artery jailing. Additionally, we plan to assess the impact of PDA stenting on PBF using machine learning, focusing on predicting metrics like the pulmonary-to-systemic flow ratio (Qp/Qs)—a critical indicator for cyanosis management in congenital heart disease [38]. Our long-term vision is to develop a predictive platform that supports clinical decision-making by optimizing procedural planning, improving efficiency, and minimizing complications and the need for reinterventions (Fig. 3). This integration aims to enhance patient outcomes by providing clinicians with data-driven insights for more precise and personalized treatment strategies.

## Conclusion

This study successfully developed a patient-specific simulation pipeline for PDA stenting, incorporating stent/balloon bending according to the guidewire pathway, accurately replicating real-world outcomes. Our approach offers significant improvements in procedural accuracy and efficiency, though future work is needed to expand the patient sample size, identify metrics associated with post-procedural complications, and further refine predictive capabilities. Ultimately, our proposed platform has the potential to improve clinical decision-making and reduce complications in PDA stenting interventions.

## Supplementary Information

Below is the link to the electronic supplementary material.

Supplementary file1 (AVI 41163 KB)

Supplementary file2 (AVI 108622 KB)

## Data Availability

Data is provided within the manuscript or supplementary information files.
